# Extracellular-vesicle containing miRNA-503-5p released by macrophages contributes to atherosclerosis

**DOI:** 10.18632/aging.103855

**Published:** 2021-04-19

**Authors:** Yuquan Wang, Zhengmin Xu, Xiaoli Wang, Jiankang Zheng, Lihan Peng, Yunfei Zhou, Yongyan Song, Zhan Lu

**Affiliations:** 1 Department of Cardiology, Affiliated Hospital of North Sichuan Medical College, Nanchong 637000, P. R. China; 2 Department of Pharmacy, North Sichuan Medical College, Nanchong 637000, P. R. China; 3 Department of Thoracic Surgery, Dazhou Central Hospital, Dazhou 635000, P. R. China; 4 School of Preclinical Medicine, and Nanchong Key Laboratory of Metabolic Drugs and Biological Products, North Sichuan Medical College, Nanchong 637000, P. R. China

**Keywords:** atherosclerosis, macrophage, extracellular vesicle, microRNA-503-5p, endothelial cells

## Abstract

Endothelial dysfunction, and the differentiation of smooth muscle cells (SMCs) into proliferative, secretory phenotypes, are two major pathophysiological processes in atherosclerosis. SMCs have the potential to recruit macrophages in atherosclerotic plaques, in which macrophages drive inflammatory responses. In this study, we found that microRNA-503-5p (miR-503-5p) was enriched in either extracellular vesicles (EVs), secreted by oxidized low-density lipoprotein-treated macrophages, or the EVs from peripheral blood mononuclear cells of atherosclerosis patients. miR-503-5p was transferred intercellularly from macrophages to the co-cultured human coronary artery endothelial cells (HCAECs) and HCASMCs *via* EVs, thus reducing the proliferative and angiogenic abilities of HCAECs and accelerating the proliferative and migrating abilities of HCASMCs. Smad family members 1, 2 and 7 were negatively regulated by miR-503-5p in HCAECs and HCASMCs. miR-503-5p was verified as an enhancer of inflammatory cytokines and adhesion molecules released by macrophages, in part *via* the down-regulation of smad family members 1, 2 and 7. The inhibition of miR-503-5p by lentivirus reduced atherosclerotic lesion formations in the aorta of atherosclerotic mice. Our work demonstrated a miR-503-5p- and EV-mediated mechanism for macrophage communication with HCAECs and HCASMCs in atherosclerosis. miR-503-5p is pro-atherosclerotic stimuli that may be a therapeutic target for atherosclerosis treatment.

## INTRODUCTION

Atherosclerosis is a disease caused by lipid-evoked inflammatory responses of vascular walls, orchestrated by the complicated interactions among different types of cells including: endothelial cells (ECs), smooth muscle cells (SMCs) and macrophages [1]. Due to facilitated roles of low endothelial shear stress produced by turbulent blood flow in atherosclerosis formation, ECs could affect atherosclerosis formation *via* affecting endothelial shear stress [2]. It has been reported that atherosclerosis is initiated by the intimal retention and modification of cholesterol-loaded lipoproteins, followed by the secretions of ECs and SMCs, recruitment of monocytes, and accumulation of inflammatory cells, extracellular matrix and lipids in the tunica intima [3]. Oxidized low-density lipoprotein (ox-LDL) is a well-known risk factor for atherosclerosis progression [4]. Ox-LDL regulates the adherence activity between endothelial cells and monocytes, which recruit monocyte-secreted macrophages into vessel walls and contribute to foam cell formation of macrophages to affect the progression of atherosclerosis [5]. Macrophages, which are scattered around the sites of plaque build-up, play vital roles in the initiation and progression of all atherosclerosis [6]. Thus, it is imperative to further investigate the underlying mechanisms of interactions among macrophages, ECs, and SMCs in atherosclerosis.

Extracellular vesicles (EVs) are lipid-bilayer heterogeneous particles of 20 nm to 2 μm in size, are involved in atherosclerosis-related processes, such as endothelial dysfunction, inflammation of vascular wall, and vascular remodeling [7]. EVs can be transferred between cells and encompass proteins, messenger RNAs (mRNAs), and microRNAs (miRNAs), and they can be secreted from ECs, SMCs, and macrophages to influence the development of atherosclerosis [8]. miRNAs refer to small non-coding RNA molecules, which serve as regulators of protein expression *via* the degradation of targeted mRNA or the blockade of protein translation [9]. A recent study has demonstrated that miRNAs are able to modulate cellular and molecular processes in the development of atherosclerosis [10]. He et al. have revealed that miR-155 is transferred to human monocytic THP1 cells with EVs as cargos to exert effects on the progression of atherosclerosis [1]. The roles of macrophage-derived EVs carrying miR-503 in atherosclerosis have also been demonstrated in previous evidence [11]. In addition, microarray analysis in the present study has demonstrated that miR-503-5p could target smad7 and smurf1/smurf2. Accumulating evidence has confirmed that miR-503 serves as a regulator in smurf2 expression and the transforming growth factor-β (TGF-β) signaling pathway [12]. TGF-β, induced by inflammatory cells, including macrophages and SMCs, is implicated in atherosclerosis [13, 14]. Smad7 is regarded as a downstream suppressive Smad in TGF-β signaling and participates in atherosclerosis by affecting a series of biological processes, including fibrosis and inflammation [15]. However, the roles of macrophage-derived EVs carrying miR-503-5p in atherosclerosis *via* Smad7-Smurf1/Smurf2-TGF-β axis have not been investigated yet. Therefore, we constructed models with macrophages treated with ox-LDL and ApoE^-/-^ mice fed with high-fat diet to explore the specific regulatory mechanism of EVs-containing miR-503-5p from macrophages as a mean to communicate with ECs and SMCs in the biology of atherosclerosis.

## RESULTS

### Expression patterns of miR-503-5p, smad7, smurf1, and smurf2 in plasma-EVs of patients with atherosclerosis

**Figure 1:**
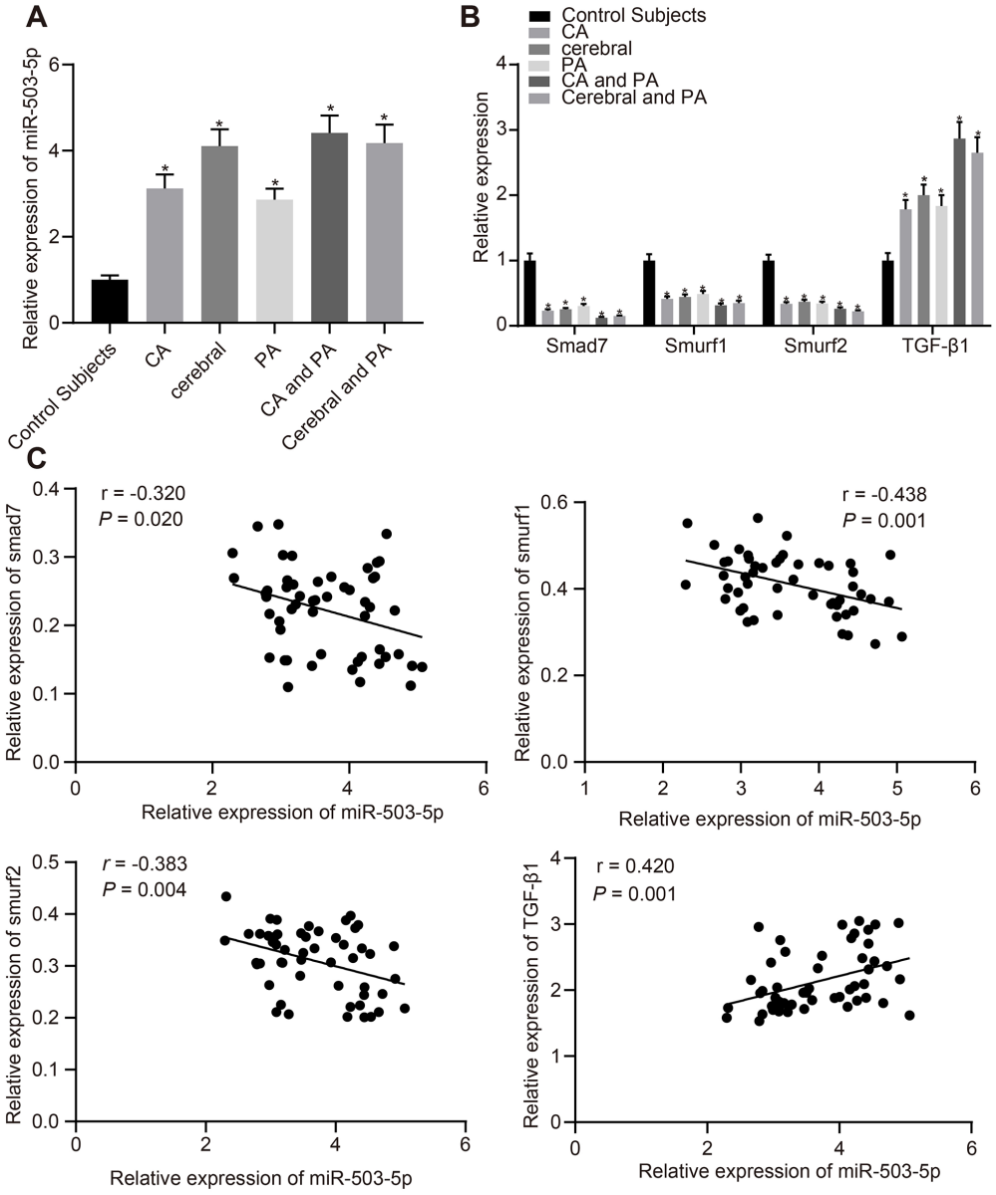
**Expression patterns of miR-503-5p, smad7, smurf1, and smurf2 in plasma-EVs of patients with atherosclerosis.** (**A**, **B**) Expression of miR-503-5p (normalized to U6), TGF-β1, smad7, smurf1, and smurf2 (all normalized to GAPDH) in plasma-EVs of healthy individuals (n = 30) and patients with atherosclerosis (n = 53) determined by RT-qPCR. (**C**) Pearson analyses of miR-503-5p and TGF-β1, smad7, smurf1, or smurf2. Values obtained from three independent experiments in triplicate were analyzed by one-way ANOVA followed by Tukey's post hoc test among three or more groups. *p < 0.05 compared with healthy individuals.

We first collected detailed information of patients with atherosclerosis (recorded in Table 1). Plasma was collected from patients with atherosclerosis and healthy individuals to determine the expression of miR-503-5p, smad/TGF-β signaling pathway molecules (smad7, smurf1, smurf2, and TGF-β1) using reverse transcription-quantitative polymerase chain reaction (RT-qPCR). The findings displayed that compared with healthy individuals, the expression of miR-503-5p and TGF-β1 was increased at different degrees in patients with coronary atherosclerosis, cerebral atherosclerosis, peripheral atherosclerosis, combined coronary atherosclerosis and peripheral atherosclerosis, and combined cerebral atherosclerosis with peripheral atherosclerosis, while the expression of smad7, smurf1, and smurf2 was decreased at different degrees (*p* < 0.05) (Figure 1A, 1B), which showed a certain linear correlation (Figure 1C).

**Table 1:** Clinical data collected from patients with AS.

**Index**	**AS group (n = 53)**	**Expression of miR-503-5p**	***p***
Age			0.598
>45	38	3.702 ± 0.681	
≤45	15	3.583 ± 0.860	
Sex			0.984
Male	29	3.670 ± 0.741	
Female	24	3.666 ± 0.730	
Risk factors			
Hypertension			0.047
Yes	10	3.258 ± 0.747	
No	43	3.764 ± 0.699	
Diabetes			0.004
Yes	11	3.124 ± 0.281	
No	42	3.811 ± 0.745	
Hyperlipemia			0.010
Yes	9	4.228 ± 0.624	
No	44	3.554 ± 0.701	
High HCY			0.004
Yes	22	3.330 ± 0.528	
No	31	3.908 ± 0.763	
Smoking			0.006
Yes	16	4.077 ± 0.766	
No	37	3.492 ± 0.646	
AS			0.001
CA	15	3.121 ± 0.327	
Cerebral	11	3.977 ± 0.378	
PA	10	2.864 ± 0.254	
CA and PA	5	4.513 ± 0.406	
Cerebral and PA	12	4.388 ± 0.392	

AS, atherosclerosis; Control Subjects, healthy individuals; CA, coronary atherosclerosis; cerebral, cerebral atherosclerosis; PA, Peripheral atherosclerosis.

### miR-503-5p had the ability to regulate human coronary artery (HCA) ECs and SMCs

**Figure 2:**
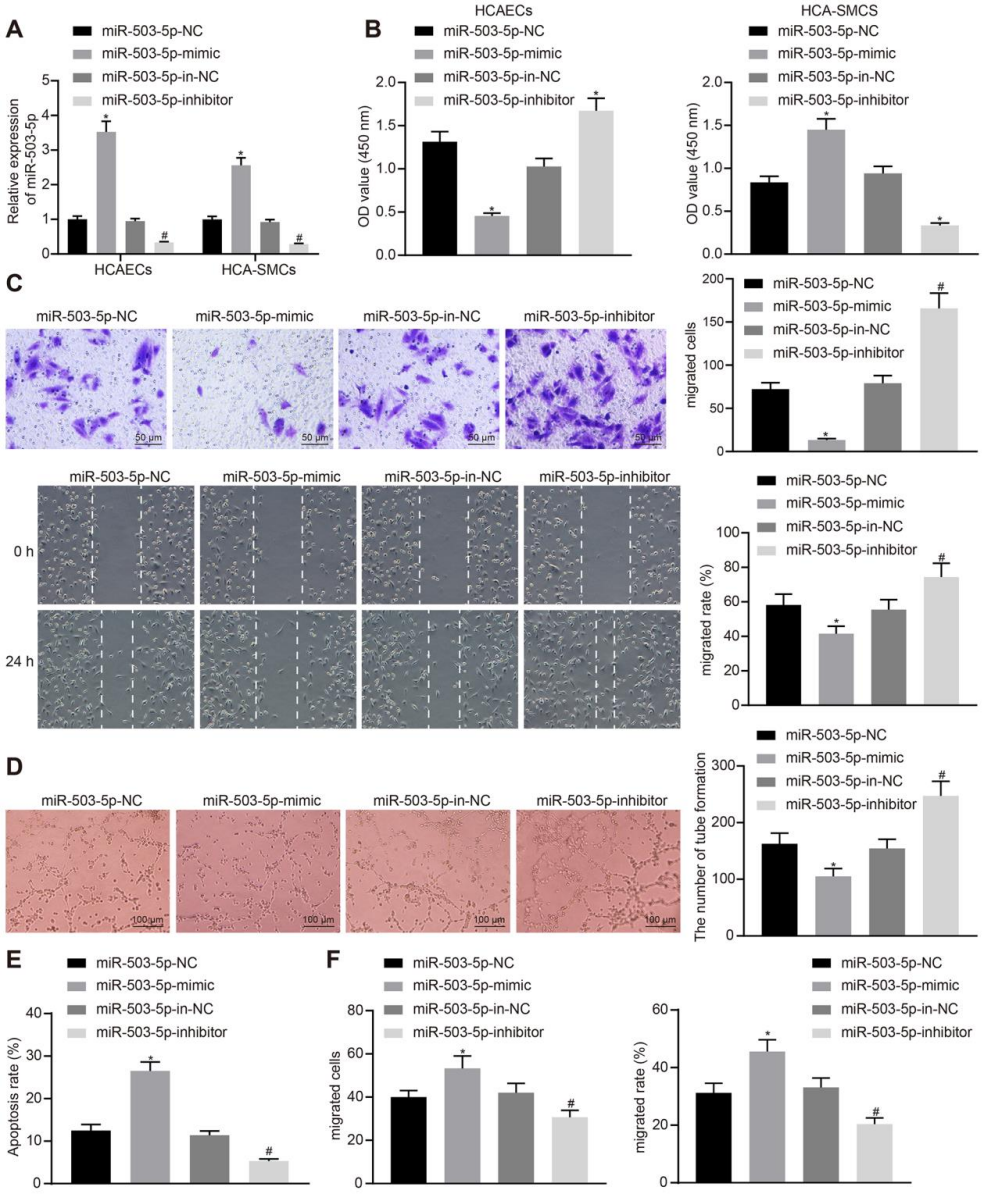
**miR-503-5p inhibits proliferation, migration, and angiogenic abilities of HCAECs while promoting the proliferation and migration of HCASMCs.** HCAECs and HCASMCs were treated with exogenous miR-503-5p mimic (with miR-mimic NC as a control) or exogenous miR-503-5p inhibitor (with miR-inhibitor NC as a control). (**A**) The expression of miR-503-5p (normalized to U6) in HCAECs and HCASMCs determined by RT-qPCR. (**B**) The proliferation of HCAECs and HCASMCs detected by CCK-8 assay. (**C**) The migration of HCAECs detected by transwell migration assays and scratch test. (**D**) Vessel-like tubes formed in HCAECs detected by Matrigel-based angiogenesis assays. (**E**) Apoptosis of HCAECs detected by flow cytometry. (**F**) The migration of HCASMCs detected by transwell migration assays and scratch test. Values obtained from three independent experiments in triplicate were analyzed by one-way ANOVA followed by Tukey's post hoc test among three or more groups. *p < 0.05 compared with HCAECs or HCASMCs treated with miR-mimic NC; *p < 0.05 compared with HCAECs or HCASMCs treated with miR-inhibitor NC.

In order to study the effects of miR-503-5p on HCAECs and HCASMCs, HCAECs and HCASMCs were initially treated with exogenous miR-503-5p mimic, exogenous miR-503-5p inhibitor, miR-mimic negative control (NC), and miR-inhibitor NC. RT-qPCR showed increased expression of miR-503-5p in HCAECs and HCASMCs treated with exogenous miR-503-5p mimic, and decreased expression of miR-503-5p in HCAECs and HCASMCs treated with exogenous miR-503-5p inhibitor (*p* < 0.05) (Figure 2A). Cell counting kit-8 (CCK-8), transwell chamber assays, scratch test, Matrigel-based angiogenesis assays, and flow cytometry revealed that the overexpression of miR-503-5p could suppress the proliferative, migrating, and angiogenic abilities of HCAECs and promote their apoptosis, while enhancing proliferation and migration of HCASMCs (*p* < 0.05). However, the downregulation of miR-503-5p exerted reverse effects (*p* < 0.05) (Figure 2B–2F). These results suggested the suppressive effects of miR-503-5p on proliferation, migration, and angiogenesis of HCAECs and promotive effects of miR-503-5p on proliferation and migration of HCASMCs.

### miR-503-5p downregulated smad7, smurf1/smurf2 and reduced the ubiquitination of TGF-β1 receptor

**Figure 3:**
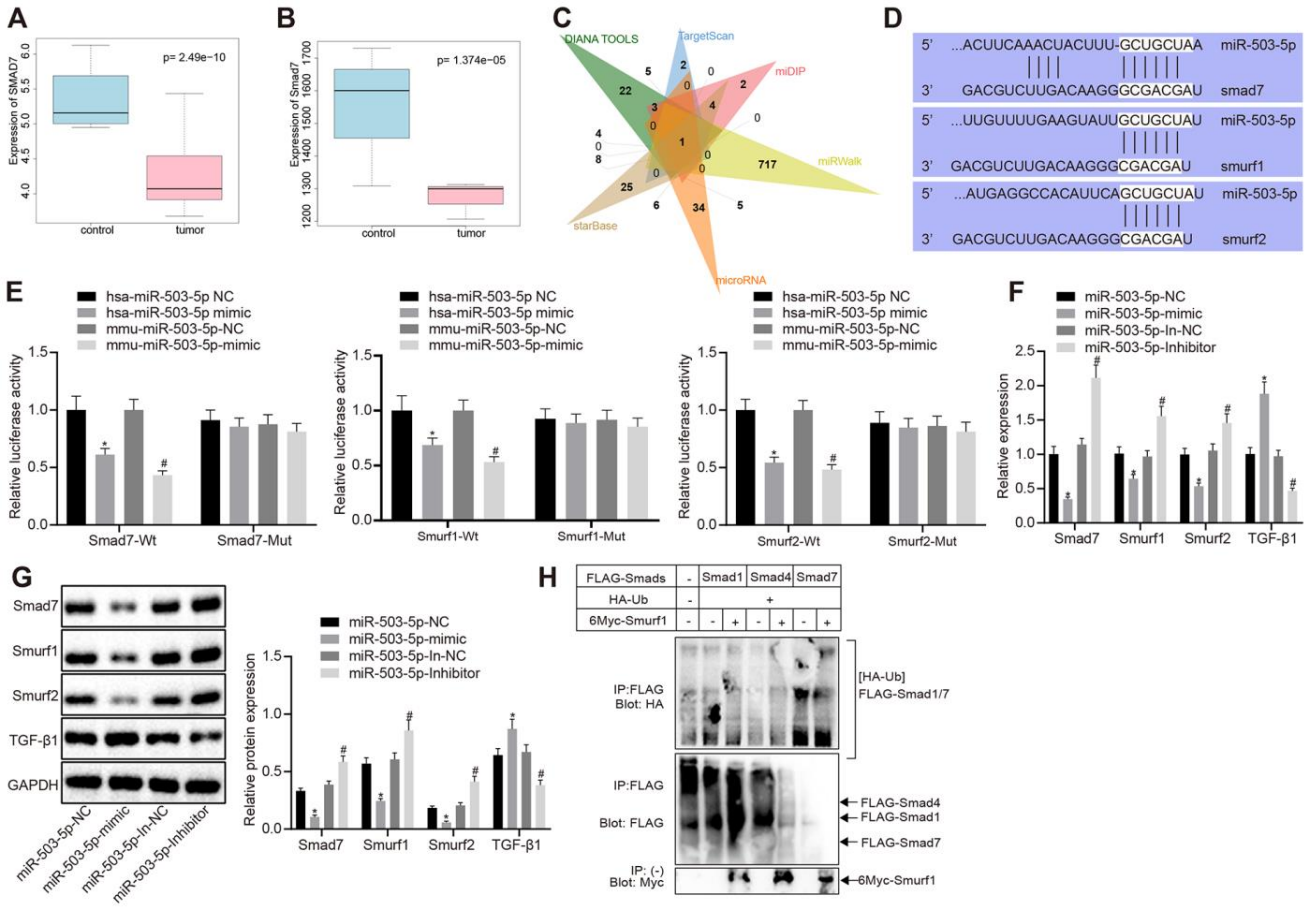
**miR-503-5p negatively regulates expression of smad7 and smurf1/smurf2 and reduces the ubiquitination of TGF-β1 receptor.** (**A**) Box plots of smad7 expression in human atherosclerosis-related microarray data GSE9490 consisted of 4 normal samples without DL-homocysteine and 8 DL-homocysteine-induced atherosclerosis samples. The blue box on the left indicates the smad7 expression of normal samples, while the red box on the right indicates the smad7 expression of diseased samples. (**B**) Box plots of smad7 expression in mouse atherosclerosis-related microarray data GSE2372. There were 3 samples obtained from the WT mice and 3 samples from ApoE mice with atherosclerosis. The blue box on the left indicates the smad7 expression in normal samples, while the red box on the right indicates the smad7 expression in diseased samples. (**C**) Venn map of predicted upstream miRNAs of smad7 in the DIANA TOOLS (miTG score > 0.90; http://diana.imis.athena-innovation.gr/DianaTools/index.php), TargetScan (context++ score percentile ≥ 99; http://www.targetscan.org/vert_71/), miDIP (Integrated Score > 0.60; http://ophid.utoronto.ca/mirDIP/), miRWalk (|energy| ≥ 25; http://mirwalk.umm.uni-heidelberg.de/), microRNA, and starBase (pancancerNum ≥ 5; http://starbase.sysu.edu.cn/). (**D**) Prediction binding site of miR-503-5p in smad7, smurf1, and smurf2 3'-UTR analyzed by TargetScan software. (**E**) Detection of luciferase activity using dual-luciferase reporter gene assay. In panels (**F**–**H**), HCAECs were treated with exogenous miR-503-5p mimic (with miR-mimic NC as control) or exogenous miR-503-5p inhibitor (with miR-inhibitor NC as control). (**F**) The mRNA levels of TGF-β1, smad7, smurf1, and smurf2 (normalized to GAPDH) in HCAECs determined by RT-qPCR. (**G**) Protein levels of TGF-β1, smad7, smurf1, and smurf2 (normalized to GAPDH) in HCAECs determined by Western blot analysis. (**H**) Effects of Smad7 on the ubiquitination of TGF-β 1 by Smurf1 and Smurf2 detected by IP assay. Values obtained from three independent experiments in triplicate were analyzed by one-way ANOVA followed by Tukey's post hoc test among three or more groups. *p < 0.05 compared with HCAECs or HCASMCs treated with miR-mimic NC; *p < 0.05 compared with HCAECs or HCASMCs treated with miR-inhibitor NC.

The microarray data GSE9490, downloaded from GEO database (https://www.ncbi.nlm.nih.gov/gds/?term=), were analyzed to screen the differentially expressed genes (|logFC| > 1, *p* < 0.05). smad7 was downregulated in DL-homocysteine-induced atherosclerosis samples when compared with the normal samples (*p* < 0.05) (Figure 3A). R language was utilized to identify the selected gene expression in mouse atherosclerosis-related microarray data GSE2372 (|logFC| > 1, *p* < 0.05). There were 6 samples in microarray data GSE2372, which contained three samples from WT mice and three samples from ApoE knockdown mice with atherosclerosis. Smad7 was also downregulated in ApoE knockdown atherosclerosis models (*p* < 0.05) (Figure 3B). Moreover, there are few studies focusing on the regulation mechanism of smad7 in atherosclerosis. DIANA TOOLS, TargetScan, miDIP, miRWalk, microRNA, and starBase database showed 48, 17, 38, 734, 66, and 60 upstream miRNAs that could target smad7, respectively. The Venn map displayed that miR-503-5p was one of the most critical upstream miRNAs in smad7 (Figure 3C). Kyoto Encyclopedia of Genes and Genomes analysis using DAVAID (https://david.ncifcrf.gov/home.jsp) revealed that smad7 could be only enriched in the TGF-β signaling pathway (hsa04350). DAVAID also presented that Smurf1 and Smurf2 could be enriched in the TGF-β signaling pathway; thus, smad7-Smurf1/Smurf2-TGF-β signaling pathway may potentially exist.

A Dual luciferase reporter gene assay was performed to verify whether smad7, smurf1, and smurf2 were target genes of miR-503-5p. The results showed that the luciferase activity of wild-type (WT)-smad7, smurf1, and smurf2 3’-UTR was significantly inhibited by miR-101-3p (*p* < 0.05), but no difference was found in mutant-type (MUT)-smad7, smurf1, and smurf2 3’-UTR (*p* > 0.05) (Figure 3E), suggesting that miR-503-5p could specifically bind to smad7, Smurf1, and Smurf2 genes. RT-qPCR revealed that mRNA levels of smad7, smurf1, and smurf2 were lowered and the TGF-β1 mRNA levels were increased in the HCAECs treated with exogenous miR-503-5p mimic (*p* < 0.05) (Figure 3F), which indicated that miR-503-5p negatively modulated the expressions of smad7, smurf1, and smurf2. Moreover, Western blot analysis displayed that the overexpression of miR-503-5p reduced protein levels of smad7, smurf1, and smurf2 and elevated TGF-β1 protein level. By contrast, the results were opposite after the depletion of miR-503-5p (*p* < 0.05) (Figure 3G), suggesting that miR-503-5p repressed the activity of smad7, smurf1, and smurf2. HCAECs were later co-transfected with Flag-labeled Smad7 (6myc-Smad7), 6myc-smurf1 or 6myc-smurf2 and HA-Ub, Flag-TGF-β1 to further prove whether the degradation of TGF-β1 was induced by the ubiquitination of Smad 7 and Smurf 1/2. IP assay revealed that smad7 could promote the ubiquitination of TGF-β1 by smurf1/2 (*p* < 0.05) (Figure 3H). Therefore, the obtained data suggested that miR-503-5p could target smad7, smurf1, and smurf2, and negatively regulate their expressions.

### Expression pattern of miR-503-5p in macrophage-EVs in the context of atherosclerosis

**Figure 4:**
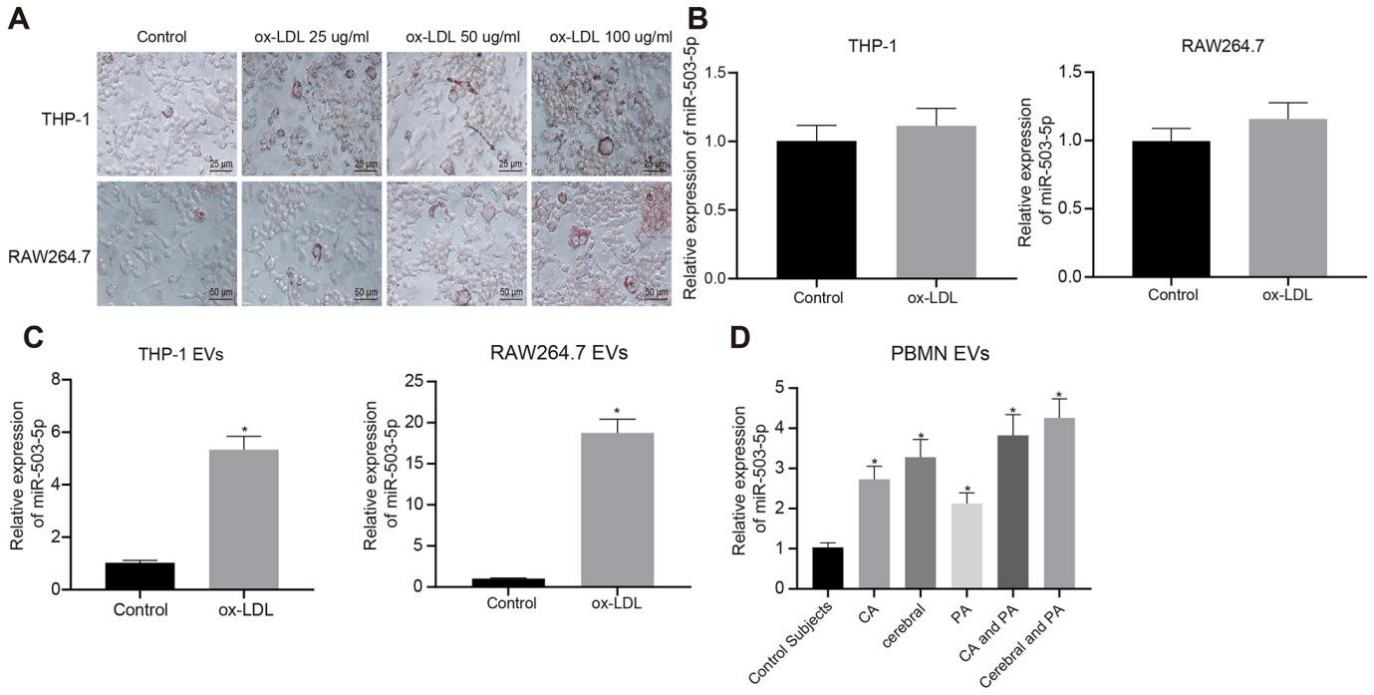
**Expression pattern of miR-503-5p in macrophage-EVs in the context of atherosclerosis.** PMA-induced THP-1 and RAW264.7 cells were treated with ox-LDL at doses of 25 μg/mL, 50 μg/mL and 100 μg/mL, respectively. (**A**) Foam cells (×200) in PMA-induced THP-1 and RAW264.7 cells detected by Oil red O staining. (**B**) Expression of miR-503-5p (normalized to U6) in PMA-induced THP-1 with or without ox-LDL treatment and their derived EVs determined by RT-qPCR. (**C**) Expression of miR-503-5p (normalized to U6) in PMA-induced RAW264.7 cells with or without ox-LDL treatment and their derived EVs determined RT-qPCR. (**D**) The expression of miR-503-5p (normalized to U6) in plasma-EVs of healthy individuals (n = 30) and patients with atherosclerosis (n = 53) determined by RT-qPCR. Values obtained from three independent experiments in triplicate were analyzed by unpaired t test between two groups and by one-way ANOVA followed by Tukey's post hoc test among three or more groups. *p < 0.05 compared with healthy individuals or PMA-induced THP-1 or RAW264.7 cells without ox-LDL treatment.

In order to explore the role of miR-503-5p in macrophage-EVs of atherosclerosis model, PMA-induced THP-1 (100 ng/mL, 24 h) or RAW264.7 cells were treated with ox-LDL (25 μg/mL, 50 μg/mL and 100 μg/mL). Oil red O staining showed an increase in staining intensity in a concentration-dependent manner, suggesting that atherosclerosis models were successfully constructed after treatment with ox-LDL (Figure 4A).

The ox-LDL concentration of 50 μg/mL was used for the following experiments. RT-qPCR showed that expression of miR-503-5p was increased in EVs from the macrophages treated with ox-LDL (*p* < 0.05) (Figure 4B), and no significant differences were found in RAW264.7 cells (*p* > 0.05) (Figure 4C). We also extracted peripheral blood mononuclear cells from healthy individuals and patients with atherosclerosis to induce macrophages, and then detected miR-503-5p expression in their derived EVs, which presented that miR-503-5p expression was increased in the EVs (*p* < 0.05) (Figure 4D).

### Macrophages delivered miR-503-5p to HCAECs and HCA-SMCs *via* EVs

**Figure 5:**
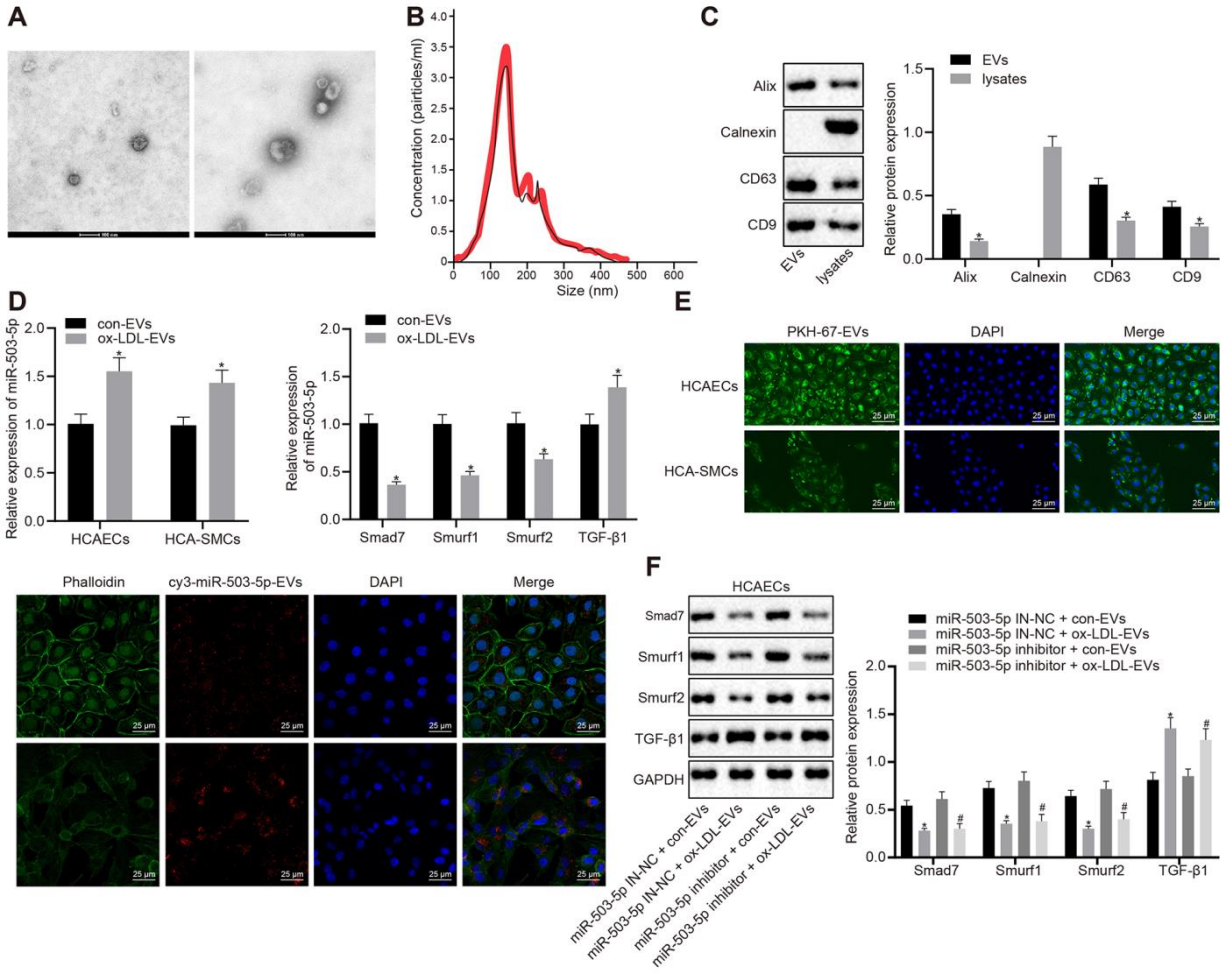
**Delivery of miR-503-5p to HCAECs and HCASMCs by macrophage-derived EVs.** (**A**) Structure and diameter of EVs observed by TEM (×100000). (**B**) Diameter and number of EVs measured by NTA. (**C**) Expression of EV marker proteins Alix, CD63, and CD9 determined by Western blot analysis; *p < 0.05 compared with EVs. HCAECs and HCASMCs were co-cultured with EVs derived from RAW264.7 cells with or without ox-LDL treatment. (**D**) Expression of miR-503-5p (normalized to U6), TGF-β1, smad7, smurf1, and smurf2 (all normalized to GAPDH) in HCAECs and HCASMCs determined RT-qPCR; *p < 0.05 compared with EVs derived from RAW264.7 cells without ox-LDL treatment. (**E**) EVs were phagocytosed by HCAECs and HCASMCs, observed under laser confocal microscope. PKH67-labeled EVs was green, DAPI-stained nuclei was blue, while cy3-miR-503-5p-labeled EVs was red (×400). (**F**) Protein expression of TGF-β1, smad7, smurf1, and smurf2 (normalized to GAPDH) in HCAECs determined by Western blot analysis. Values obtained from three independent experiments in triplicate were analyzed by unpaired t test between two groups and by one-way ANOVA followed by Tukey's post hoc test among three or more groups. *p < 0.05 compared with miR-inhibitor NC-treated HCAECs co-cultured with EVs in the absence of ox-LDL; # p < 0.05 compared with miR-503-5p inhibitor treated HCAECs co-cultured with EVs in the absence of ox-LDL.

In order to study the mechanism of macrophage-EVs in HCAECs and HCASMCs *in vitro*, the supernatants of RAW264.7 macrophages treated with ox-LDL and that of the normal control were collected to isolate EVs. A transmission electron microscope (TEM) displayed the structure and diameter of EVs (Figure 5A) and Nanoparticle tracking analyzer (NTA) revealed diameter and number of EVs (Figure 5B). Western blot analysis showed the presence of EV marker proteins: Alix, CD63, and CD9 in EVs but the presence of calnexin only in cells (Figure 5C). We further validated the functional changes of macrophage-EVs co-cultured with HCAECs or HCASMCs, and the specific downstream mechanisms of HCAECs *in vitro*. HCAECs and HCASMCs were co-cultured with EVs from ox-LDL-treated macrophages and control macrophages, namely ox-LDL-EVs and con-EVs. RT-qPCR indicated that the expressions of miR-503-5p and TGF-β1 were elevated and the expressions of smad7, Smurf1, and smurf2 were decreased in HCAECs and HCASMCs co-cultured with ox-LDL-EVs (*p* < 0.05) (Figure 5D). Confocal scanning laser microscopic imaging revealed that EVs were internalized by HCAECs or HCASMCs and distributed around the nucleus (Figure 5E). Macrophages delivered cy3-miR-503-5p to HCAECs or HCASMCs *via* EVs (*p* < 0.05) (Figure 5F). Western blot analysis presented that TGF-β1 protein levels were elevated and protein levels of smad7, Smurf1, and smurf2 were reduced in HCAECs co-cultured with ox-LDL-EVs and treated with lentivirus-mediated depleted miR-503-5p (*p* < 0.05) (Figure 5G). The data above elucidated that macrophages delivered miR-503-5p to HCAECs and HCASMCs *via* EVs that may affect functions of HCAECs and HCASMCs.

### Macrophage-EVs carrying miR-503-5p affects the functions of HCAECs and HCASMCs

**Figure 6:**
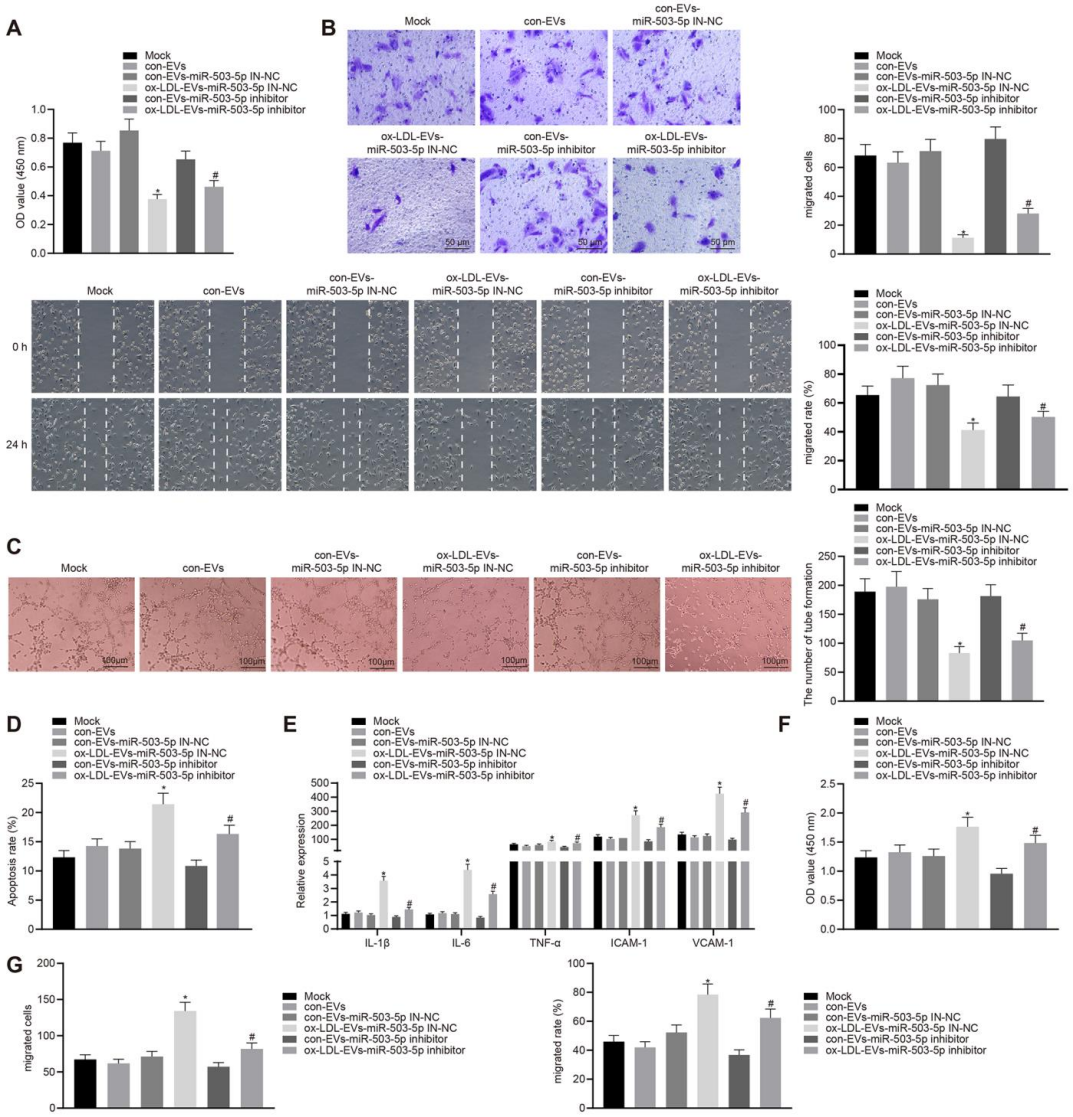
**Macrophage-EVs carrying miR-503-5p inhibit proliferation, migration, and angiogenic abilities of HCAECs while promoting proliferation and migration of HCASMCs.** HCAECs and HCASMCs treated with miR-503-5p inhibitor (with miR-503-5p in-NC as control) were co-cultured with EVs released by RAW264.7 cells with or without ox-LDL treatment. (**A**) Proliferation of HCAECs detected by CCK-8 assay. (**B**) Migration of HCAECs detected by transwell migration assays and scratch test. (**C**) Vessel like tubes formed in HCAECs detected by Matrigel-based angiogenesis assays; Scale bar = 20 μm. (**D**) Apoptosis of HCAECs detected by flow cytometry. (**E**) Expression of pro-inflammatory factors (IL-1β, IL-6, and TNF-α) and adhesion molecules (ICAM-1 and VCAM-1) measured by ELISA methods. (**F**) Proliferation of HCASMCs detected by CCK-8 assay. (**G**) Migration of HCASMCs detected by transwell migration assays and scratch test. Values obtained from three independent experiments in triplicate were analyzed b y oneway ANOVA followed by Tukey's post hoc test among three or more groups. *p < 0.05 compared with miR-inhibitor NC-treated HCAECs co-cultured with EVs in the absence of ox-LDL; # p < 0.05 compared with miR-503-5p inhibitor-treated HCAECs co-cultured with EVs in the absence of ox-LDL.

HCAECs co-cultured with ox-LDL-EVs displayed inhibited proliferative, migrative, and angiogenic capabilities, and promoted apoptosis and secretion of inflammatory factors and adhesion molecules (*p* < 0.05) (Figure 6A–6F). On the contrary, the depletion of miR-503-5p could reverse the aforementioned changes. HCASMCs co-cultured with ox-LDL-EVs showed increased proliferation and migration and that the downregulation of miR-503-5p could reverse the facilitative roles of ox-LDL-EVs in the proliferation and migration of HCASMCs (*p* < 0.05) (Figure 6G–6I).

### miR-503-5p regulated the functions of HCAECs and HCASMCs by downregulating Smad7, smurf1, and smurf2

**Figure 7:**
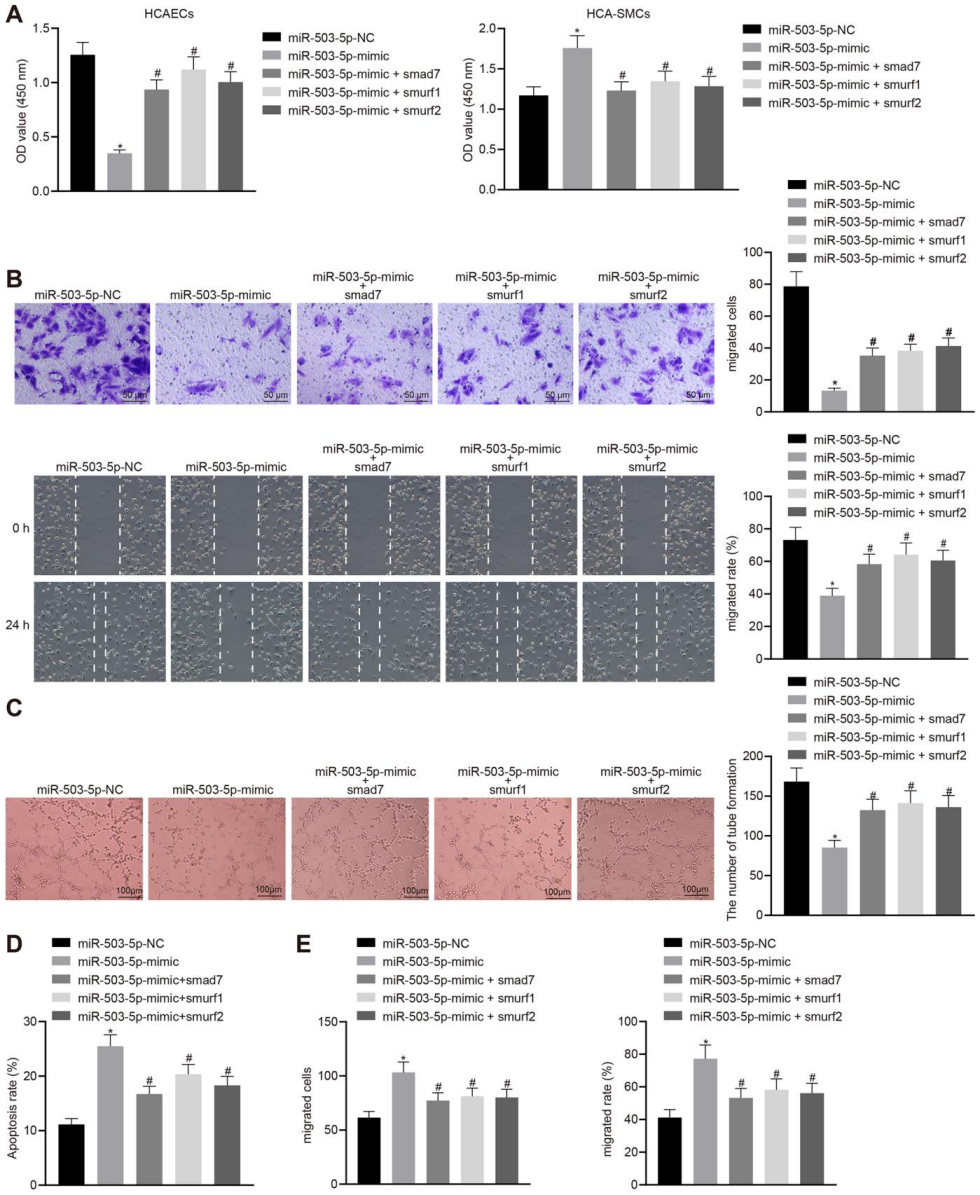
**Overexpression of Smad7, smurf1, and smurf2 abrogates the effects of miR-503-5p on HCAECs and HCASMCs.** HCAECs and HCASMCs were treated with exogenous miR-503-5p mimic (with miR-mimic NC as control) and expression vectors containing smad7, smurf1, or smurf2 gene. (**A**) Proliferation of HCAECs and HCASMCs detected by CCK-8 assay. (**B**) Migration of HCAECs detected by transwell migration assays and scratch test (×200). (**C**) Vessel-like tube-forming ability in HCAECs detected by Matrigel-based angiogenesis assays (×100). (**D**) Apoptosis of HCAECs detected by flow cytometry. (**E**) Migration of HCASMCs detected by transwell migration assays and scratch test (×200). Values obtained from three independent experiments in triplicate were analyzed by one-way ANOVA followed by Tukey's post hoc test among three or more groups. * *p* < 0.05 compared with HCAECs or HCASMCs treated with miR-mimic NC; # *p* < 0.05 compared with miR-503-5p mimic.

HCAECs and HCASMCs were treated with exogenous miR-503-5p mimic and the expression vector containing smad7, smurf1, or smurf2 gene to explore the effects of miR-503-5p, Smad7, smurf1, and smurf2 in the cellular biology of HCAECs and HCASMCs. Overexpression of miR-503-5p could suppress proliferating, migrating, and angiogenic capabilities, and induce apoptosis of HCAECs (*p* < 0.05) (Figure 7A–7D), while facilitating proliferation and migration of HCASMCs (*p* < 0.05) (Figure 7A, 7E). HCAECs and HCASMCs that were treated with the expression vector containing smad7, smurf1, or smurf2 gene could reverse these changes (*p* < 0.05) (Figure 7A–7E). Therefore, the down-regulation of Smad7, smurf1, and smurf2 conferred the effects of miR-503-5p on the functions of HCAECs and HCASMCs.

### Pro-atherosclerotic role of miR-503-5p in the formation of atherosclerotic plaques

**Figure 8:**
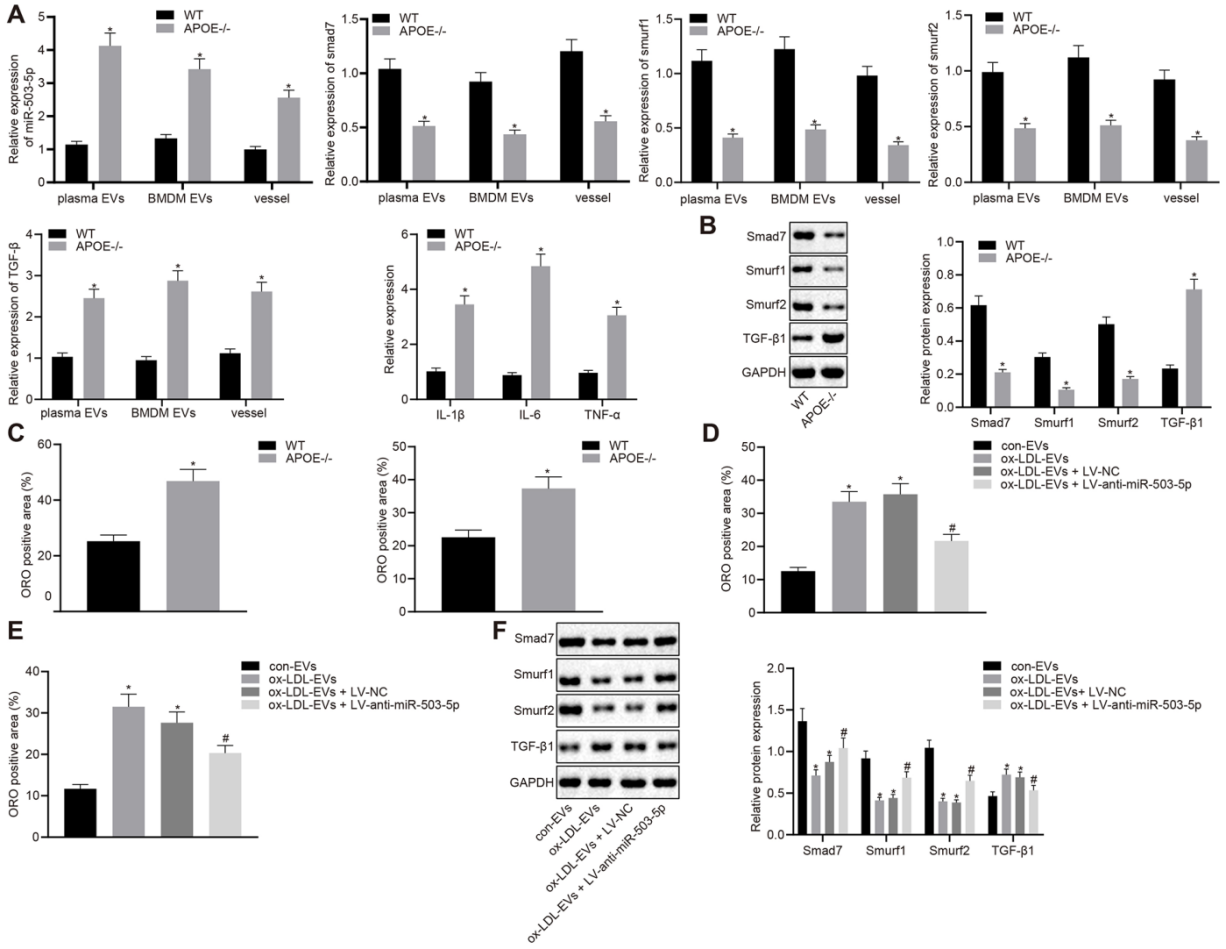
**Depletion of miR-503-5p inhibits the formation of atherosclerotic plaques *in vivo*.** (**A**) Expression of miR-503-5p (normalized to U6), and TGF-β1, smad7, smurf1, and smurf2 (normalized to GAPDH) in plasma-EVs, macrophage-EVs, and arterial tissues of ApoE-/- mice (n = 3) and WT mice (n = 3) determined by RT-qPCR. (**B**) Protein levels of TGF-β1, smad7, smurf1, and smurf2 (normalized to GAPDH) in plasma-EVs, macrophage-EVs, and arterial tissues of ApoE-/- mice (n = 3) and WT mice (n = 3) determined by Western blot analysis. (**C**) Lipid deposition in mouse arterial tissues detected by Oil red O staining; Scale bar = 50 μm. (**D**) EVs derived from macrophages with or without ox-LDL treatment were separately injected into the tail vein of C57BL6 WT mice at dose of 100 μg for each. One week later, EVsinjected mice were injected with lentivirus expressing anti-miR-503-5p and anti-miR-503-5p NC. (**E**) The lipid plaque of arterial tissues of C57BL/6J WT mice (n = 12) detected by Oil red O staining. (**F**) Expression of smad7, smurf1, and smurf2 (normalized to GAPDH) in arterial tissues of C57BL/6J WT mice (n = 12) determined by Western blot analysis. Values obtained from three independent experiments in triplicate were analyzed by unpaired t test between two groups and by one-way ANOVA followed by Tukey's post hoc test among three or more groups. * p < 0.05 compared with WT mice or mice injected with EVs derived from macrophages without ox-LDL treatment; # p < 0.05 compared with mice injected with EVs derived from macrophages with ox-LDL treatment followed by lentivirus expressing anti-miR-503-5p NC.

RT-qPCR was lastly adopted to determine the levels of miR-503-5p, smad7, smurf1, smurf2, IL-1β, IL-6, and TNF-α in plasma-EVs, macrophage-EVs, and vascular tissues of ApoE^-/-^ mice (Figure 8A). The results revealed that the expressions of miR-503-5p and TGF-β1 were elevated in plasma-EVs, macrophage-EVs, and vascular tissues of ApoE^-/-^ mice, while the expressions of smad7, smurf1, and smurf2 were decreased. Meanwhile, Western blot analysis showed reduced protein levels of smad7 and smurf1/2 in mouse arterial tissues but increased TGF-β protein levels in the vascular tissues of ApoE^-/-^ mice (*p* < 0.05) (Figure 8B). Oil red O staining showed increased lipid deposition in mouse arterial tissues, which aggravated the progression of atherosclerosis (*p* < 0.05) (Figure 8C). To further verify the pro-atherosclerotic role of miR-503-5p in the formation of atherosclerotic plaques in mice, C57BL/6J WT mice were injected with EVs derived from macrophages with or without ox-LDL treatment, followed by injection with lentivirus expressing anti-miR-503-5p and anti-miR-503-5p NC. Oil red O staining (Figure 8D) and Western blot analysis (Figure 8E) demonstrated that depleted levels of miR-503-5p reduced lipid deposition, but increased the expressions of smad7, smurf1, and smurf2 in mouse arterial tissues (*p* < 0.05). These findings proved that the expression of miR-503-5p was upregulated in atherosclerosis and the inhibition of miR-503-5p could subsequently hinder the progression of atherosclerosis.

## DISCUSSION

Atherosclerosis is one of the pathophysiological factors that contribute to acute myocardial infarctions and cerebrovascular accidents and is also the leading cause of cardiovascular deaths worldwide [16]. In recent years, it has been found that EVs-derived miRNAs from macrophages serve as mediators in atherosclerosis [17]. Our study is aimed at exploring the promoting effects of EV-carrying miR-503-5p from macrophages on the proliferation and migration of HCAECs and HCASMCs in atherosclerosis. Taken together, this study reveals that macrophage-derived EVs carrying miR-503-5p can inhibit proliferation, migration, and angiogenic ability of HCAECs, while promoting the proliferation and migration of HCASMCs by downregulating Smad7, smurf1, and smurf2 and elevating TGF-β1, thus aggravating atherosclerosis.

Our results demonstrated that miR-503-5p was enriched in plasma-EVs and macrophage-EVs in atherosclerosis. The aberrant expression of miRNAs has a clearly established role in the process of atherosclerosis [18]. miR-150 is demonstrated to be highly expressed in PMA-induced THP-1 cells in atherosclerosis [19]. Nguyen et al. have indicated that several miRNAs, including miR-503, are abundantly expressed in macrophage-derived EVs in atherosclerosis [17], which is consistent with our findings. Moreover, bioinformatics analysis, in combination with the dual luciferase reporter gene assay validated that miRNA-503-5p could target and negatively regulate smad7, smurf1, and smurf2, being enriched in the TGF-β signaling pathway. The TGF-β signal is dysregulated in the development of atherosclerosis [13]. smad7 expression has been demonstrated to display a reduction in atherosclerosis [15]. Cao et al. have revealed that downregulated levels miR-503 could elevate Smurf2 levels by regulating TGF-β/Smad2 signaling [12]. Also, miR-503 could target smad7 and regulate its expression *via* TGFβ1 signaling pathway [20], which will be further elucidated through the following serial experiments.

In addition, the data in present study demonstrates that macrophages were able to upregulate miR-503-5p and transfer miR-503-5p to HCAECs and HCASMCs with EVs as cargos. EVs remain small membrane vesicles, including exosomes that are released during the exocytosis of multivesicular bodies, and sprouting from plasma membrane as well [21]. It is also interesting to note that EVs participate in cell-to-cell communication, as they harbor lipids, proteins, mRNAs and miRNAs [7]. Moreover, EVs derived from VECs, VSMCs, and macrophages could transfer multiple molecules to the recipient cells in atherosclerosis [8]. EVs contain many miRNAs that can be delivered to ECs and SMCs to affect the development of cardiovascular diseases [22]. Most importantly, our findings revealed that macrophages could deliver miR-503-5p to HCAECs and HCASMCs to exert promotive effects on the process of atherosclerosis. Based on the *in vitro* and *in vivo* experiments, the present study presented that macrophage-secreted EVs carrying miR-503-5p inhibits proliferation, migration, and angiogenic potentials of HCAECs, while accelerating the proliferation and migration of HCASMCs in atherosclerosis *via* TGF-β/smad7/smurf1/smurf2 axis. Atherosclerotic plaques are prone to rupture, and angiogenesis is a source of intraplaque hemorrhage [16]. ECs have the ability to transform into smooth muscle-like cells in atherosclerosis, and SMCs could differentiate into proliferative, secretory phenotypes and form macrophage/foam cells in atherosclerosis [3]. Hergenreider et al. have confirmed that the protective stimulation of atherosclerosis secretes communicating signals between ECs and SMCs through a miRNA- and EVs-mediated mechanism, which provides a potential target for treatment of atherosclerosis [2]. Studies have revealed that THP-1 monocytes-derived miR-150 into ECs *via* EVs could promote migration and angiogenic ability of ECs and inhibit apoptosis in atherosclerosis [22, 23]. A recent study has demonstrated that miR-155 is enriched in ox-LDL-induced ECs-derived EVs and then transferred to THP1 cells, by which EVs could induce inflammatory responses [1]. Upregulated miRNAs in thermogenic macrophage-derived EVs could aggravate the development of atherosclerosis by inhibiting the migration of ECs and enhancing macrophage enrichment in vessel walls [17]. The downregulation of miR-34a could promote viability and inhibit apoptosis of ox-LDL-treated human aortic endothelial cells by upregulating Bcl-2, thus indicating a promising biomarker for atherosclerosis therapy [24]. The proliferation of vascular smooth muscle cells (VSMCs) is involved in the process of atherosclerosis, and elevated expressions of miR-29b induced by ox-LDL could facilitate VSMC migration [25]. Upregulated miR-503 could also promote SMC differentiation by directly targeting smad *via* TGFβ1 signaling pathways [20]. Herein, it was reasonable to conclude that macrophages delivering miR-503-5p *via* EVs possessed a great therapeutic potential in atherosclerosis.

**Figure 9:**
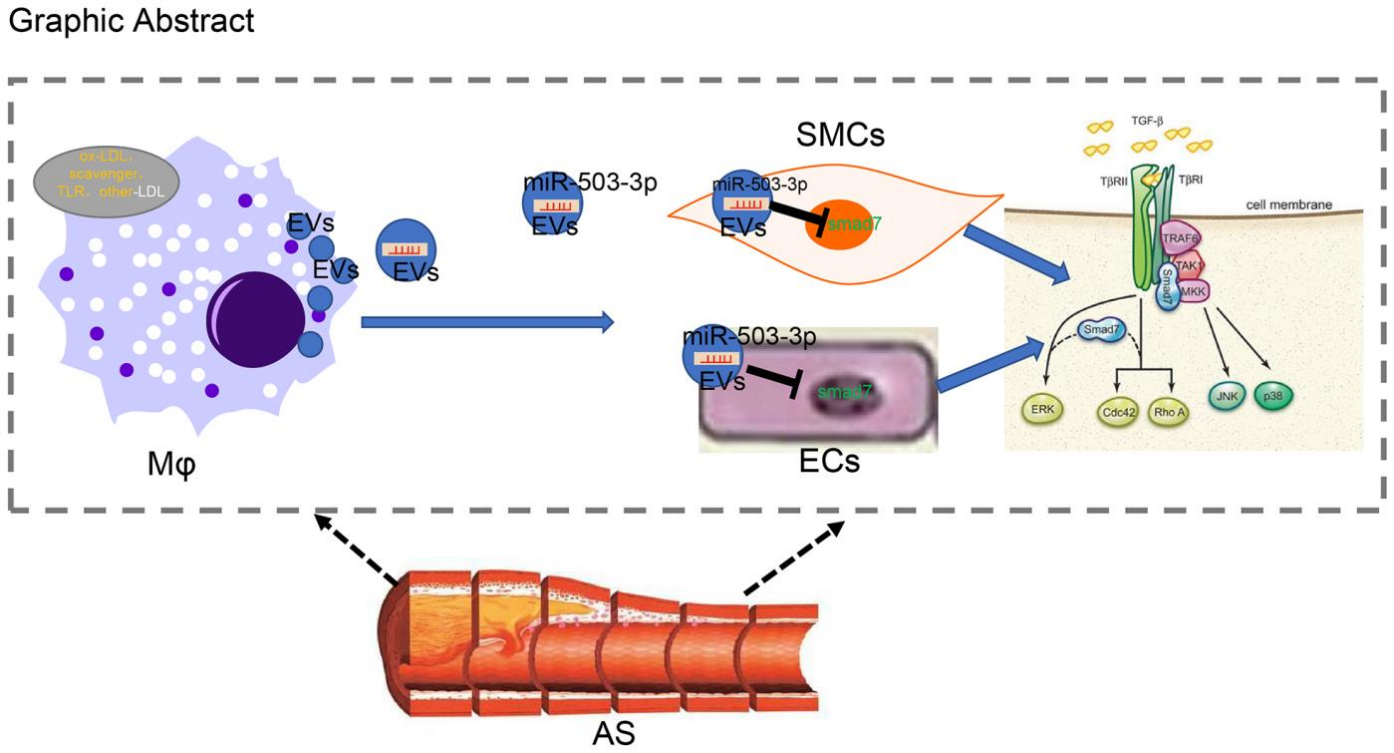
**Systematic diagrams illustrating how macrophages crosstalk with ECs and SMCs and how they involve in the formation of atherosclerotic plaques.** Macrophages deliver miR-503-5p to HCAECs and HCA-SMCs *via* EVs and downregulate Smad7, smurf1, and smurf2 in HCAECs and HCASMCs, thereby inhibiting proliferation, migration, and angiogenic abilities of HCAECs, but promoting proliferation and migration of HCASMCs.

In conclusion, we have demonstrated that macrophages can secrete EVs and transmit miR-503-5p into HCAECs and HCASMCs in order to inhibit the proliferative and angiogenic functions of HCAECs, while inhibiting the proliferation and migration of HCASMCs (Figure 9). Consequently, anti-miR-503-5p was validated to exert an atheroprotective effect in the murine model. Thus, EVs from macrophages with upregulated levels of miR-503-5p may function as a promising new therapeutic target for atherosclerosis. However, the research is still in the preclinical stage, and the investigation on the mechanism of action is still insufficient. Future experiments are needed in order to further explore the intrinsic mechanisms.

## MATERIALS AND METHODS

### Ethics statement

The Ethics Committee of the Affiliated Hospital of North Sichuan Medical College approved the study protocol. Informed written consent was obtained from each patient prior to the study. Animals were treated in strict accordance with the Guide for the Care and Use of Laboratory animals published by the US National Institutes of Health (NIH). Extensive efforts were made to minimize the number of animals used in the experiments and their suffering.

### Study subjects

Human monocyte cell line THP-1, RAW264.7 mouse cell line (monocyte-macrophage leukemia cells), human coronary artery endothelial cells (HCAECs) and human coronary artery smooth muscle cells (HCASMCs) were purchased from American Type Culture Collection (ATCC, Manassas, VA, USA). The recovered THP-1 were cultured with Royal Park Memorial Institute (RPMI) 1640 medium (Gibco Company, Grand Island, NY, USA), supplemented with 10% fetal bovine serum (FBS) and 1% streptomycin/penicillin. The THP-1 cells were then induced into macrophages using 100 ng/ml PMA (Sigma-Aldrich Chemical Company, St Louis, MO, USA), and RAW264.7 cells were cultured with Dulbecco’s modified Eagle’s medium (DMEM) (Gibco Company, USA) containing 10% FBS and 1% penicillin/streptomycin. HCAECs were incubated with EBM-2 (containing cytokine) containing 5% FBS (Lonza Group Ltd., Basel, Switzerland)., and HCASMCs were incubated with SMC-GM. All cells were cultured in an incubator (Thermo Fisher Scientific, Rockford, IL, USA) at 37° C with 5% CO_2_. Peripheral blood samples were collected from healthy individuals as previously described [26]. The peripheral blood mononuclear cells were separated from the peripheral blood using monocyte separation solution Histopaque® 1077 density gradient (Sigma-Aldrich, USA) *via* centrifugation. After treatment with ox-LDL (Yeasen Company, Shanghai, China) at different concentrations for 24 hours, THP-1-derived macrophages were utilized to construct atherosclerosis models.

### Cell treatment

HCAECs and HCASMCs were treated with miR-503-5p mimic, miR-503-5p inhibitor, expression vectors containing full fragments of smad7 gene, Smurf1 gene, or smurf2 gene to manipulate the expression of miR-503-5p, smad7, Smurf1 and smurf2. Mimic NC, inhibitor NC and empty vector were utilized for corresponding control. Macrophages were treated with Cy3-miR-503-5p mimic (Shanghai GenePharma Co., Ltd., Shanghai, China) and incubated in the serum-free medium. Transient transfection was achieved using Lipofectamine 3000 (Invitrogen, at#L3000015) following the protocol provided by the manufacturer.

### Dual luciferase reporter gene assay

Dual luciferase reporter gene assay was performed to identify whether smad7, smurf1, and smurf2 were direct target genes of miR-503-5p. The 3'UTR gene fragments of smad7, smurf1, and Smurf2 were artificially synthesized and subsequently introduced into pGL3-control vectors (Promega, Madison, WI, USA) using XhoI and NheI restriction endonuclease sites. Mutation sites of complementary sequences of seed sequences were designed based on smad7, Smurf1 and Smurf2 WT plasmids. The verified pGL3-WT or pGL3-MUT of smad7, smurf1, smurf2 were co-transfected with miR-503-5p mimic and mimic NC into HEK293 cells from Shanghai Institute of Biochemistry and Cell Biology, Chinese Academy of Sciences (Shanghai, China). Dual Luciferase Reporter Assay System kit (Promega, USA) was employed to detect the luciferase activity on the TD-20/20 Luminometer (E5311, Promega, USA).

### RNA isolation and quantitation

Total RNA was extracted from macrophage-EVs and plasma-EVs using TRIzol (15596026, Thermo Fisher, MA, USA). Next, the extracted total RNA was reverse-transcribed into cDNA according to the instructions of the Reverse transcription Kit (Takara Bio Inc., Otsu, Shiga, Japan). RT-qPCR was carried out with a mixture of 2 μL reverse transcription reagent, 10 uL TB Green Premix Ex Taq II (Takara, Shiga, Japan), and 10 μM primer reactions were run on a LightCycler 480 (Roche). U6 was used as the internal reference for miR-503-5p in cells, and cel-miR-39 as the external reference for miR-503-5p in EVs. The expression ratio of the target gene between the experimental and control groups was calculated using the 2^-ΔΔCt^ method. Primers are listed in Table 2.

**Table 2:** Primer sequences for RT-qPCR.

**Target**	**Primer sequences (5’-3’)**
hsa-miR-503-5p RT-primer	GTCGTATCCAGTGCAGGGTCCGAGGTATTCGCACTGGATACGAC
hsa-miR-503-5p	F: TAGCAGCGGGAACAGTTCTGCAG
	R: GTGCAGGGTCCGAGGT
U6	F: CTCGCTTCGGCAGCACA
	R: AACGCTTCACGAATTTGCGT
Human smad7	F: CCTTAGCCGACTCTGCGAACTA
	R: CCAGATAATTCGTTCCCCCTGT
Human Smurf1	F: CTGGATGCTTTTGGTCTGGT
	R: CCTGATAGACGCGAACACAG
Human Smurf2	F: CGATGGCTGTTAGCAGCTTTTC
	R: 5'-TGCCTCTGCAGGGCTTCAAAG
Human TGF-β	F: CGATGGCTGTTAGCAGCTTTTC
	R: TGCCTCTGCAGGGCTTCAAAG
Human GAPDH	F:CATCGAAGGTGGAAGAGTGG
	R: CATCAAGAAGGTGGTGAAGC
Cel-miR-39-3p	F: GGTCACCGGGTGTAAATCAGCTTG
Mmu-miR-503-5p	F:TAGCAGCGGGAACAGTACTGC
Mmu-U6	F: CTCGCTTCGGCAGCACA
Mmu-Smurf1	F: AGTTCGTGGCCAAATAGTGG
	R: GTTCCTTCGTTCTCCAGCAG
Mmu-Smurf2	F: GTGAAGAGCTCGGTCCTTTG
	R: AGAGCCGGGGATCTGTAAAT
Mmu-smad7	F: GTGGCATACTGGGAGGAGAA
	R: GATGGAGAAACCAGGGAACA
Mmu-TGF-β	F: ACTGCTATGCTGCCTGCTCTTACT
	R: TGGCCTTGTAGACACCTTGGTCTT
Mmu-GAPDH	F: CATCAAGAAGGTGGTGAAGC
	R: CATCGAAGGTGGAAGAGTTGG

F, forward; R, reverse

### Western blot analysis

Cell lysates were collected from lysis buffer (Beyotime Institute of Biotechnology, Shanghai, China) containing phosphatase inhibitors, protease inhibitors and phenylmethylsulfonyl fluoride. The proteins (15-20 μg) were subjected to 30% Acrylamide-Bis gel (8%-12%), and transferred onto the polyvinylidene fluoride membrane (0.22 μm) (Merck Millipore, Billerica, MA, USA). The membrane was incubated with the following primary antibodies: calnexin (Cat.No. 10427-2-AP, Proteintech Group Inc., IL, USA), β-actin (ab8226, 1: 1000, Abcam, UK), CD63 rabbit antibody (1: 1000, ab134045, Abcam, Cambridge, UK), TSG101 rabbit antibody (1: 1000, ab125011, Abcam), CD81 rabbit polyclonal antibody (1: 1000, ab109201, Abcam), Alix rabbit antibody (1 μg/ml, ab76608, Abcam) (1: 1000), smad7 (R&D systems, MAB2029, 1: 1000), SMURF1 (ab236081, 1:1000), SMURF2 [EP629Y3] (ab53316, 1:1000), TGF beta 1 (ab92486, 1:1000), phospho (p)-JNKThr183/Tyr185 (cst4668, 1:1000), p-p38 MAPK (Thr180/Tyr182) (cst4511, 1:1000), p38 (cst8690, 1:1000), and JNK (cst9252, 1: 1000). β-actin was used as the internal reference. Results were visualized with horseradish peroxidase-coupled anti-rabbit immunoglobulin (Dako) using enhanced chemilumine-scence detection reagents. Band densities of targeted proteins were analyzed by Quantity One (Bio-Rad) analysis software and normalized to that of β-actin.

### Isolation and characterization of macrophage-EVs

The EVs were isolated by ultracentrifugation. Briefly, macrophages were cultured with conditioned medium (DMEM + 10% EV-depleted serum or RPMI 1640 + 10% EV-depleted medium) for 48 h and then were centrifuged at 500 × g for 15 min, 2000 × g for 15 min, and at 10,000 × g for 20 min. The macrophages were finally filtered through 0.22 μm filter two times, followed by ultracentrifugation at 110, 000 ×g for 70 min.

Macrophage-EVs were further purified by density gradient ultracentrifugation of iodixanol. EVs were placed on the 12-floor OptiPrep (Sigma-Aldrich, USA) gradient consisted of 30, 27.5, 25, 22.5, 20, 17.5, 15, 12.5, 10, 7.5, 5, and 2.5% iodixanol in 20 mM Hepes (pH 7.2), 150 mM NaCl, 1 mM Na_3_VO_4_, and 50 mM NaF. Macrophage-EVs were then centrifuged using a SW40Ti rotor (Beckman Coulter; Indianapolis, IN) at 110,000 × g at 4° C for 16 h, followed by ultracentrifugation at 110,000 × g for 70 min at 4° C. As previously mentioned, approximately 2 × 10^5^ macrophages were treated with 2 ug exosome protein.

NTA was used to measure the number and diameter of EVs. The 20 μg EVs were dissolved in 1 mL PBS and whirled for 1 min to maintain homogeneity. A NanoSight NTA (Malvern Panalytical, UK) system was subsequently used to analyze EV distribution.

The fresh EV samples (20 μL) were loaded on the carbon-coated copper electron microscopy grids for 2 min with under the guidance of a TEM, and labeled with phosphotungstic acid solution (12501-23-4, Sigma-Aldrich, USA) or negative staining for 5 min. The grids were washed with PBS three times to remove the remaining phosphotungstic acid solution, and semi-dried with filter paper. The images were observed under the Hitachi H7650 TEM (80KV, Hitachi, Tokyo, Japan).

### Macrophage-EV uptake assay

The PKH67 green fluorescence was used for labeling the purified macrophage-EVs and macrophage-EVs carrying Cy3-microRNA-503-5p mimic according to the manufacturer’s instructions (Sigma Aldrich, USA). PKH67-labeled EVs were co-cultured with HCAECs or HCASMCs for 1 h, followed by the fixation in 4% paraformaldehyde and staining with Phalloidin-iFLour 594 (Abcam, UK) for 30 min. The nuclei were stained with 4', 6-diamino-2-phenylindole (DAPI). The internalization of EVs by HCAECs and HCASMCs was observed under microscope or confocal microscope (Carl Zeiss MicroImaging, Inc., Thornwood, NY, USA).

### CCK-8 assays

HCAECs and HCASMCs were seeded into 96-well plates at a density of 3000 cells/well (100 μL), with or without extracted macrophage-EVs at a concentration of 10 μg/mL, 20 μg/mL, and 40 μg/m. The first detection was conducted at 12^th^ h or 24^th^ h. Each well was added with 10 mL CCK-8 reagent (DOJINDO, CK04), and the plates were placed in the cell incubator and incubated for 2 h. The absorbance value of each well was measured on a microplate reader at 450 nm [27].

### Flow cytometric analysis

The Annexin V-APC apoptosis detection kit (Ebioscience, Thermo Fisher, USA) was employed to detect cell apoptosis. In brief, after transfection for 48 h, cells were collected and detached with trypsin without ethylenediaminetetracetic acid (EDTA). Cells were centrifuged and re-suspended in 200 μL binding buffer after being washed with PBS two times. The cells were later added with 10 μL fluorescein isothiocyanate (FITC)-Annexin V and 5 μL propidium iodide (PI) and mixed without light exposure at room temperature for 15 min, followed by incubation with 300 μL binding buffer. A flow cytometer (FACScan; BD Biosciences, Franklin Lakes, NJ, USA) with CellQuest software (BD Biosciences, USA) was utilized to detect apoptosis at the excitation wavelength of 488 nm and the emission wavelength of more than 630 nm.

### Transwell migration assays

Cell suspension was incubated with the serum-free medium, which was added into the apical chamber containing 3 × 10^4^ cells/100 ul (3422, Corning). The basolateral chamber was added with 700 uL 10% FBS/medium with or without 20 μg/ml EVs, either derived from untreated or ox-LDL-treated macrophages. The chamber was cultured in an incubator for 12-16 h (smooth muscle cells usually left to incubate for 12 h, while endothelial cells generally for 16 h). The basolateral chamber was then fixed in 95% ethanol for 30 min, and the cells were stained with 4 g/L crystal violet (Sigma-Aldrich, USA) for 30 min under the microscope.

### Scratch test

The scratches were made on HCAECs and HCASMCs using a 20-200 μL pipette and were left to incubate in the serum-free medium with or without 20 ug/ml EVs, either derived from untreated or ox-LDL-treated macrophages. The cells were photographed at 0 h, and further cultured in an incubator for 24 h. Finally, the migration of cells was analyzed following photograph.

### Matrigel-based angiogenesis assays *in vitro*

A total of 3 × 10^4^ serum-free medium-incubated HCAECs were seeded in 96-well plates coated with Matrigel (50 ul/well, 356234, Corning) with or without 20 μg/ml EVs, either derived from untreated or ox-LDL-treated macrophages. After 8 h, vessel-like tubes formed in cells were photographed and observed and analyzed using Image J.

### Enzyme-linked immunosorbent assay (ELISA)

The contents of inflammatory cytokines IL-1β, TNF-α, IL-6; and adhesion molecules ICAM-1, VCAM-1 in HCAECs were measured using an ELISA kit (Shenzhen Dako Biological Co., Ltd., Shenzhen, China) following the instructions as provided.

### C57BL6 WT and ApoE^-/-^ mice

A total of 12 C57BL6 WT and 12 ApoE^-/-^ male mice (aged 6-8 weeks and weighing 18-24 g) were purchased from the Laboratory Animal Center of Peking University, and fed in specific-pathogen-free environment in the Laboratory Animal Center of Affiliated Hospital of North Sichuan Medical College. All mice were euthanized by cervical dislocation after anesthesia induction. The blood vessels of mice were collected with sodium citrate anticoagulant tubes. The EVs were extracted according to the abovementioned methods. The plasma-EVs and EVs derived from mouse bone marrow derived macrophages were extracted. Mice were given a high-fat diet (21% crude fat, 0.15% cholesterol, and 19.5% casein) for 12 weeks [28].

### Lentivirus transduction *in vivo*

The plasmids, anti-miR-503-5p and anti-miR-503-5p NC, (Exiqon, Vedbaek, Denmark) (each 16 μg) and two envelope plasmids, PSPAX2 (12 μg) and PMD2G (4.8 μg), were co-transfected into HEK293T cells using Lipofectamine 3000 (at#L3000015, Invitrogen) according to the instructions provided by manufacturer. Forty-eight hours later, the supernatant was concentrated using a Centricon Plus-70 filter (UFC910096, Millipore, USA). Lentivirus at titer of 10^8^ TU/mL was used in the experiment. EVs derived from macrophages with or without ox-LDL treatment were separately injected into the tail vein of C57BL6 WT mice at dose of 100 μg for each. One week later, EV-treated mice were injected with lentivirus expressing anti-miR-503-5p and anti-miR-503-5p NC for once a week. After the mice were fed with high-fat diet for 12 weeks, they were euthanized by cervical dislocation for Oil red O staining and Western blot analysis.

### Oil red O staining

The mouse aorta was subject to Oil red O staining as previously described [28].

### Sample collection

The study consisted of 53 patients (29 males and 24 females) with atherosclerosis who were hospitalized in the Department of Cardiology and Department of Neurology in the Affiliated Hospital of North Sichuan Medical College from July 2018 to December 2018. Another 30 healthy individuals (17 males and 13 females) were also enrolled in this study. The inclusion criteria were as follows: a, patients with coronary atherosclerosis diagnosed by coronary angiography, b, patients with brain atherosclerosis diagnosed by CT Scanning; c, patients with peripheral atherosclerosis (carotid atherosclerosis, lower extremity atherosclerosis) diagnosed by ultrasonography. The exclusion criteria were as follows: patients with rheumatic heart disease, acute myocardial infarction, recent history of trauma surgery, medication history of oral contraceptives, folic acid, and vitamin B, renal insufficiency, blood urea nitrogen > 7.11 mmol/L, creatinine > 106 mmol/L, heart failure, various bleeding diseases or a proclivity for bleeding, poor blood pressure control [systolic pressure ≥ 160 mm Hg(1 mm Hg = 0.133 kPa) or diastolic pressure ≥ 100 mm Hg], poor heart rate control (heart rate ≥ 90 times/min), limb infection, thrombophlebitis, severe varicosities, deep vein thrombosis, severe valvular disease, congenital heart disease, aortic aneurysm, artery dissection, coronary artery fistula, coronary aneurysm, or arrhythmia with possible interference of ECG gating function of equipment. EVs were obtained by gradient centrifugation from 5-10 mL whole blood samples, which were collected from patients and healthy individuals, and placed into sodium citrate anticoagulant tubes.

### Statistical analysis

Data conforming to normal distribution were expressed as mean ± standard deviation. Unpaired *t* test was performed for statistical comparison between two groups and one-way analysis of variance (ANOVA) with Tukey’s post hoc test for statistical comparisons among multiple groups. The relationship between the two indicators was analyzed by Pearson Correlation Analysis. Statistical significance was set at a level of *p* < 0.05 and analyzed using SPSS 24.0 software (IBM Corp. Armonk, NY, USA).
